# V_2_O_5_ Thin Films as Nitrogen Dioxide Sensors †

**DOI:** 10.3390/s18124177

**Published:** 2018-11-28

**Authors:** Krystyna Schneider, Wojciech Maziarz

**Affiliations:** AGH University of Science and Technology, Faculty of Computer Science, Electronics and Telecommunications, Department of Electronics, 30-059 Krakow, Poland; maziarz@agh.edu.pl

**Keywords:** vanadium pentoxide, thin film, reactive sputtering, electrical properties, nitrogen dioxide, gas sensor, metal-insulator transition (MIT)

## Abstract

Vanadium pentoxide thin films were deposited onto insulating support by means of rf reactive sputtering from a metallic vanadium target. Argon-oxygen gas mixtures of different compositions controlled by the flow rates were used for sputtering. X-ray diffraction at glancing incidence (GIXD) and Scanning Electronic Microscopy (SEM) were used for structural and phase characterization. Thickness of the films was determined by the profilometry. It has been confirmed by GIXD that the deposited films are composed of V_2_O_5_ phase. The gas sensing properties of V_2_O_5_ thin films were investigated at temperatures from range 410–617 K upon NO_2_ gas of 4–20 ppm. The investigated material exhibited good response and reversibility towards nitrogen dioxide. The effect of metal-insulator transition (MIT) on sensor performance has been observed and discussed for the first time. It was found that a considerable increase of the sensor sensitivity occured above 545 K, which is related to postulated metal-insulator transition.

## 1. Introduction

Increasing environmental pollution is becoming a vital global concern, particularly in relation to the imperative to reduce emissions of gases causing the greenhouse effect, acid rain, and the depletion of stratospheric ozone. Therefore, there is an urgent need to develop some devices that allow fast, portable, low-cost monitoring of the gases responsible for air pollution and/or pose danger to human health. So far, sophisticated and expensive equipment, such as gas analyzers based on IR and UV spectrophotometry, pulse fluorescence, flame photometry and gas chromatography, to determine air quality were applied. Although this equipment enables very precise gas phase analysis, it has four substantial disadvantages such as high cost, large dimensions (limited portability), slow analysis time and non-continuous monitoring of the gas composition. In this respect, chemical gas sensors may offer advantages in the form of simple construction, low cost and ability to work in situ. One large group of sensors, applied to environmental monitoring, is based on liquid (or wet) electrochemistry. However, these sensors presently suffer from the same four severe disadvantages. Moreover, these sensors cannot be applied in environments warmer than room temperature.

During the past five decades, efforts were made to develop chemical gas sensors based on solid-state technology. The potential advantages of these sensors over the wet technology sensors are (i) miniaturization, (ii) simple calibration and measurement, (iii) low cost, (iv) short response time, (v) resistance to severe conditions, such as high temperature and corrosive environment and (vi) selectivity. Their main advantage is that they can also operate at elevated temperatures, thus meeting the environmental requirements, such as for instance in car and industrial exhaust systems.

There are several types of chemical gas sensors [1]. The largest group consists of electrochemical sensors. According to Wilson et al. [2] and Janata [3] the electrochemical gas sensors have been categorized into three groups: (1) conductometric (measurement of electrical conductivity or related value such as resistivity, impedance); (2) potentiometric (measurement of electromotive force or related value such as voltage of the solid cells); (3) amperometric (measurement of current).

The most widely studied type of the gas sensors are conductometric. Their principle of operation involves a change in the resistance of the sensor sensitive phase (usually metal oxide) upon exposure to a specific component of the gas atmosphere. During adsorption of gas molecules on solid surface, the electronic charges (electrons or electron holes) are created, which change surface electrical conductivity. This process takes place within the thin layer near-surface of the solid of the thickness named the Debye length. Consequently, electrical conductivity may be used as a sensing property for detection of specific gas phase components. Various review works have referred to conductometric gas sensors [4,5,6,7,8,9]. However, there is no simple theory which can predict how the sensor’s signal depends on gas concentration.

Conductometric gas sensors are usually based on metal oxide semiconductors, such as SnO_2_ [10,11,12,13,14,15], ZnO [16,17], WO_3_ [18], TiO_2_ [19,20,21], Fe_2_O_3_ [22]. Also, other materials such as graphene, metal hydroxides, metal dichalcogenides, phosphorene, boron nitride [4,5,7], and conducting polymers are investigated. However, the poor sensor recovery and stability remain the major concerns.

Generally, semiconductor gas sensors suffer from low selectivity. Several attempts were made to improve their selectivity in respect to the detectable gas. The most frequent approach involves change of the sensor composition by doping, the use of composites with one or more other materials, variation of operating temperature etc. It was found that the successful method improving sensors’ selectivity is addition of some heterogeneous catalysts, such as noble metals (Pt, Pd, Au) to the sensor material. In addition, use of the vanadium pentoxide – well-known catalyst – is promising. 

Recently, vanadium oxides have attracted considerable interest due to their multi-valence, good chemical stability and excellent catalytic properties [23]. Moreover, unlike the above- mentioned sensing oxides, vanadium oxides show metal-insulator transition (MIT), an interesting electrical property which may have impact on the sensor’s performance.

V_2_O_5_, the most stable compound among over 15 known vanadium oxides, is a promising NO_2_ sensor material [24]. It demonstrated high sensitivity and selectivity for ethanol [25], ammonia [26], hydrogen and hydrocarbons [27]. 

Table 1 [27,28,29,30,31,32,33,34,35,36,37,38,39,40,41,42,43,44,45,46,47,48,49] includes examples of conductometric sensors of various gases, except of NO_2_, with V_2_O_5_ sensing material or V_2_O_5_ as addition to other material such as SnO_2_ [33,38], TiO_2_ [40,50] and other [29,37,51,52,53,54]. On the other hand, Table 2 summarizes V_2_O_5_-based NO_2_ sensors.

For some practical applications, the high operating temperature creates problems with the long-term stability and high costs of the sensor manufacture and maintenance. As can be seen in the Table 1, some authors reported that their sensors based on nanostructured vanadium pentoxide or its composite show sensitivity at room temperature [29,31,36,41,48,49]. Moreover, as per published results [40], the sensor selectivity may be substantially improved for sensors composed of oriented nanoparticles.

In this paper, the application of V_2_O_5_ thin films as NO_2_ gas sensor is reported. The effect of MIT on sensor performance was studied for the first time. 

Nitrogen dioxide, NO_2_ is an extremely toxic gas. It is produced by all combustion in air and by industrial processes. NO_2_ is responsible for various pollution problems such as smog and acid rain. Therefore, there is an urgent need to develop devices that allow fast, portable, low-cost monitoring of the NO_2_ in the interest of environment and human health. Successful development of NO_2_ gas sensors for commercialization requires the achieving of four “S”, i.e. sensitivity, selectivity, stability and speed (response and recovery rates).

## 2. Materials and Methods

### 2.1. Thin Film Preparation

**Figure 1:**
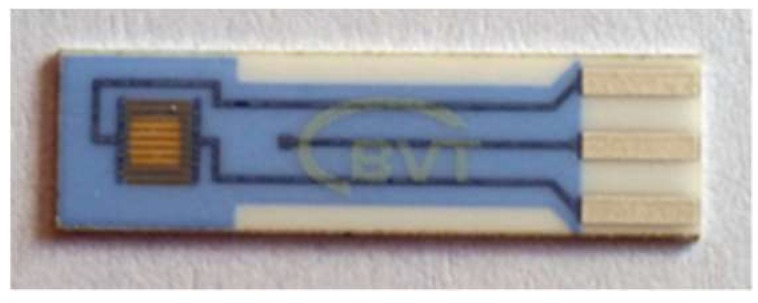
Conductometric sensor support from BVT Technologies company.

VO_x_ thin films were deposited onto insulating support (either fused silica or alumina) for sample characterization or conductometric sensor substrate type CC1.W (BVT Technologies, Czech Rep.), for electrical measurements, by means of rf sputtering in a reactive atmosphere at working pressure 4.75 Pa (24% O_2_ – 76% Ar) from a metallic V target. Conductometric supports presented in Figure 1 were provided by BVT Technologies. Details of the film deposition are given elsewhere [27].

### 2.2. Morphology and Structural Characterization

Scanning electron microscopy (SEM) studies were carried out for as-sputtered thin films using NOVA NANOSEM 200 (FEI Europe Company) microscope. Phase composition of as-sputtered thin films was studied by X-ray diffraction at glancing incidence, GIXD. Thickness of the V_2_O_5_ thin film was (210 ± 25) nm.

### 2.3. Sensing Characterization

The responses *S* of films to the target gas (NO_2_), defined as changes in electrical resistance (*S* = *R*_NO2_/*R*_air_), were measured by custom-made setup at different NO_2_ concentrations (0–20 ppm). The sample was placed in a gas chamber on a workholder, where the temperature and gas atmosphere (gas composition and humidity) were stabilized. The relative humidity was set to 50 ± 0.1%. The requested NO_2_ concentration was obtained by controlling the ratio of gas to air flow rate, and total flow rate was set to 500 cm^3^/min. The total flow rate was maintained at the same level of 500 cm^3^/min. The film resistance changes upon NO_2_ were measured with Keithley 6517 electrometer working in constant voltage mode (*U* = 0.1 V). At the beginning, the sensor response has been stabilized in pre-set conditions (at the lowest temperature, constant gas flow, pure air of 50% humidity). Then two types of measurement procedures were performed. During the first stage, the measurement of sensor response under varying temperature (range 483–623 K), and at constant NO_2_ concentration (20 ppm) fed alternately with air for purging purposes, was realized. After that, the second stage was performed: The measurement at constant temperature under increasing gas concentrations (0–20 ppm with 4 ppm step), also while purging the chamber with air. The sensor resistance was sampled every 2 s. The sensor measurements were performed within the temperature range extending from RT to 700 K. Equipment applied for measurements of the sensor characteristics was described in detail elsewhere [55].

## 3. Results and Discussion

### 3.1. Structural and Microstructural Characteristics

**Figure 2:**
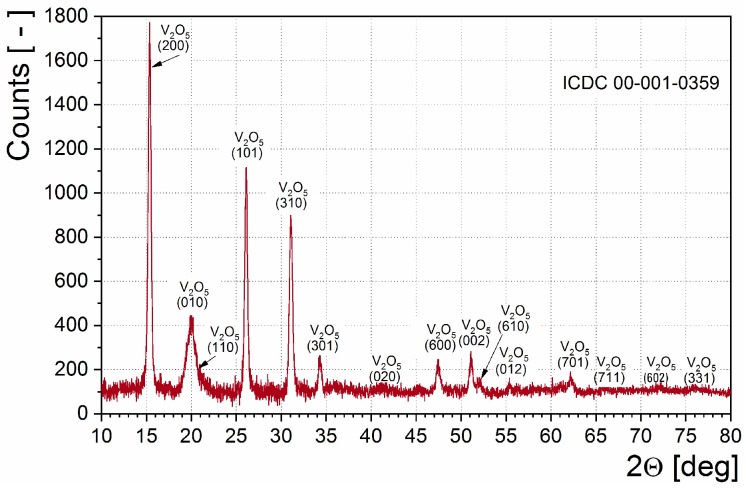
X-ray diffraction patterns for as-sputtered V_2_O_5_ thin film.

Figure 2 presents the typical XRD patterns of the sample annealed at 673 K in argon atmosphere. X-ray diffraction analysis of the samples revealed the presence of the V_2_O_5_ orthorhombic phase. 

The determined lattice parameters (*a* = 1.145 ± 0.003 nm; *b* = 0.436 ± 0.004 nm; *c* = 0.355 ± 0.003 nm) well agree with those from literature reports [56]. Presented XRD patterns were used for determination of the crystallite size. Crystallite size, *d*_XRD_, was calculated according to Scherrer’s method: *d*_XRD_ = 20.0 ± 1.8 nm. No effect of sintering temperature on obtained XRD results was observed. 

**Figure 3:**
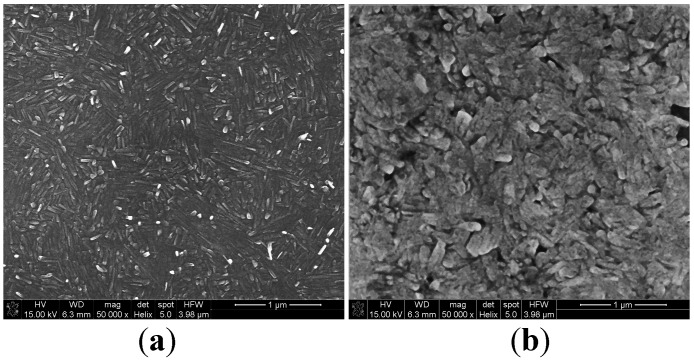
Scanning electron micrographs of: (**a**) as-sputtered thin film; (**b**) after annealing at 673 K.

As can be seen, the as-sputtered thin films (Figure 3a) are poly-dispersed, and the grains are mostly columnar in shape with a length of 565 ± 100 nm and diameter of 220 ± 40 nm. On the other hand, after sintering (Figure 3b) they are rather spherical (mean diameter = 500 ± 75 nm). Chemical analysis performed by EDS technique revealed presence of high peaks coming from the silicon support and short peaks corresponding to oxygen and vanadium elements. 

### 3.2. Sensing Characteristics

**Figure 4:**
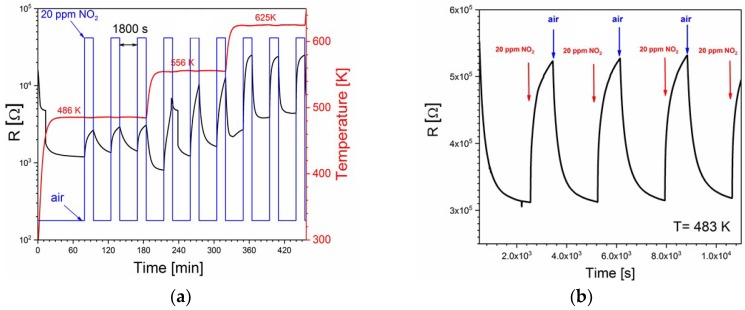
Dynamic changes in the electrical resistance of V_2_O_5_ thin film upon interaction with 20 ppm NO_2_, (**a**) at several temperatures; (**b**) at 483 K.

An example of V_2_O_5_ sensor responses to 20 ppm NO_2_ are shown in Figure 4a,b.

As one can see from Figure 4, the resistance of the investigated V_2_O_5_ thin films increases upon exposure to NO_2_, which is characteristic of n-type semiconductors.

NO_2_ sensing mechanism can be explained in terms of defect structure of vanadium pentoxide [57]. V_2_O_5_ equilibrated in air shows deficit, x, of oxygen (V_2_O_5−x_). Doubly positive ionized oxygen vacancies are compensated by V^4+^ ions. Electrical conductivity is achieved by small polaron mechanism, via electron hopping from neighbouring two ions V^4+^ and V^5+^ (marked as 1 and 2):

V^4+^_(1)_ + V^5+^_(2)_ → V^5+^_(1)_ + V^4+^_(2)_ (1)

Electrical conductivity is proportional to concentration of V^4+^ ions.

When NO_2_ is added to gas atmosphere, adsorption of NO_2_ molecules takes place according to the reaction:

(2) $$\;NO_{2\left(g\right)}+V^{4+}\Rightarrow NO_{2\left(ad\right)}^{-}+V^{5+}\;$$

Equation (2) indicates that the increase of the resistance upon exposure of NO_2_, as observed in Figure 4a,b, results from decrease of the concentration of V^4+^ ions. 

**Figure 5:**
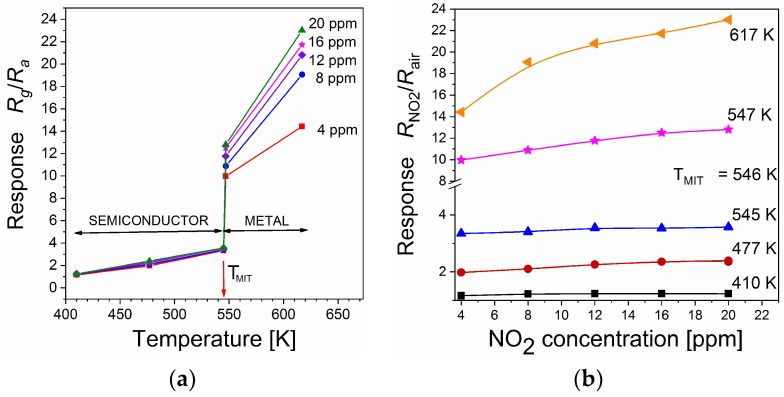
The sensor response *S*: (**a**) *vs.* temperature for various NO_2_ concentrations and (**b**) *vs.* NO_2_ concentration for different temperatures.

Figure 5a illustrates sensor response, *S = R_NO2_/R_air_*, versus temperature. The abrupt increase in the sensor response is observed at 545–547 K. This behaviour may be explained by the occurrence of the metal-insulator transition, MIT. 

One of the most spectacular phenomena occurring in most vanadium oxides is the abrupt change of the electrical resistivity from the values typical for semiconductor to those typical of metal phase. This phenomenon, called semiconductor–metal phase transition, SMPT [58] or more frequently metal-insulator transition, MIT, offers immense prospects for various practical applications, in particular oxide electronics, photoelectronics [59,60] and gas sensors. Strelcov et al. [61] studied gas sensor properties of nanowire VO_2_ close to temperature of MIT (*T*_MIT_ = 338 K). They observed that varying the temperature of the nanowire close to the *T*_MIT_, the conductance of the nanowire becomes extremely responsive to the tiny changes in the ambient gas environment. According to our knowledge, the effect of MIT on gas sensor properties of V_2_O_5_ have not been studied yet.

The single valence vanadium oxides V_2_O_3_, VO_2_, and most of double valence vanadium oxides (i.e., Magneli or Wedsley series) show metal-insulator transition. During this transition, the polymorphic phase transition occurs from lower to higher symmetrical crystallographic structure. However, vanadium pentoxide, V_2_O_5_ thin film is an exception. At temperatures close to 540 K (*T_MIT_* = 530 ± 5 K, Blum [59]; *T*_MIT_ = 553 K, Kang et al. [60]), MIT takes place in nanostructured and thin films of V_2_O_5_ without any phase transition. This fact can be explained that MIT in V_2_O_5_ is limited to the surface layer [59]. Our recent studies revealed that MIT is observed in V_2_O_5_ thin films annealed in air, while in V_2_O_5_ thin films annealed in gas atmosphere containing NO_2_ MIT effect was not observed. From these facts and Equation (2), we postulate that sufficient V^4+^ ion concentration on the surface is a prerequisite to MIT in vanadium pentoxide. The abrupt change (Figure 5a) of sensor response *S*, which is observed close to *T*_MIT_, results from MIT in sample in air (i.e. abrupt decrease of *R*_air_, and much smaller increase in *R*_NO2_.

As Figure 5b indicates, below *T*_MIT_ (*T* < *T*_MIT_ = 546 K), the sensor response *S* increases slightly with temperature. On the other hand, for *T* > *T*_MIT_ the sensor response exhibits much larger increase. 

Some examples of NO_2_ sensor with vanadium pentoxide sensing material are given in Table 2 [47,62,63,64,65]. Among gas sensing oxides presented in Table 2, two materials distinguish oneself: composite porous Si/nanorod V_2_O_5_ [51] and thin film V_2_O_5_ (this work) sensors. They enabled detection of low NO_2_ concentrations below 5 ppm. They showed the highest response *S* = 5–10 [51] and *S* = 14–23 (this work), respectively. Proposed by Yan et al. [51] sensor can work at room temperature. However, the instability of gas sensitivity, lack thermal stability of porous silicon [66], as well as complex technology of manufacturing of the porous Si/nanorod V_2_O_5_ composite [51] limit the commercial applications.

## 4. Conclusions

In the present work, we fabricated V_2_O_5_ thin films by rf reactive sputtering. The film structure and morphology were studied by X-ray diffraction at glancing incidence and scanning electronic microscopy. Gas sensing studies showed that the V_2_O_5_ thin films were sensitive to NO_2_ at relatively low operating temperatures. The considerable increase of the sensor sensitivity was observed above 545 K, which is related to postulated metal-insulator transition. Presented studies on vanadium pentoxide thin film NO_2_ sensor will be continued regarding selectivity (especially in respect to water vapor).

## Figures and Tables

**Table 1:** Vanadium oxide-based gas sensors, literature survey (published between 2010–2017).

Gas	Composition	Morphology	Operation Temp (K)	Response	Gas Concentration [ppm]	Sensitive Against	Ref.
H_2_ CH_4_ C_3_H_8_	V_2_O_5_	Thin films	420–520	1.22	5–300 H_2_ 50–3000 CH_4_, C_3_H_8_	NA	[27]
Et^1^, NH_3_	V_2_O_5_	Hollow spheres	NA^2^	Et:1.02–1.06 NH_3_:1.01–1.02	100–500	NA	[28]
NH_3_	V_2_O_5_	Thin film	RT	Change of color	100–400	H_2_	[29]
Et NH_3_	V_2_O_5_	Thin film	NA	1.04 1.06	100–500 100–500	NA NA	[30]
Acetone, CH_3_OH, HCHO, toluene	VO_2_	nanorods	RT^3^	1.015 1.027 1.060 1.055	5–100	NA	[31,32]
SO_2_	SnO_2_ + 5 wt% MgO + 2 wt% V_2_O_5_	Thick film	NA	1.44	0.1–1	NA	[33]
Et	V_2_O_5_	Thin film	508	NA	2500	NA	[34]
xylene	V_2_O_5_	Thin film	300	17 27.9	800 100	NH_3_, Et, toluene, acetone	[35]
1-butyl- amine	V_2_O_5_	Nanofibres	RT	1.42	0.15–9.5	NH_3_, propranolol, toluene	[36]
NH_3_	V_2_O_5_	Composite fibers with polyvinyl acetate and pyrrolidone	530	1.02 1.06	0.1–0.8		[37]
BTEX^4^	SnO_2_/V_2_O_5_	Composite SnO_2_/V_2_O_5_	540	5.5–6	0.5–50	Et, CH_3_OH, HCHO	[38]
CH_4_	VO_2_	Thin films	298–473	1.008–1.032	50–500	NO_2_, H_2_	[39]
NH_3_	VWT: V_2_O_5_–WO_3_–TiO_2_	Potentio- metric: Au V_2_O_5_–WO_3_– TiO_2_	820	0–150	10–300	NA	[40]
H_2_O	VO_2_ (3fl) complex	Thick film 20-30μm	RT	NA	RH: 35–70%	NA	[41]
NH_3_	V_2_O_5_–V_7_O_16_	Thin film	620	1.4	0.16–0.32	NO, CO	[42]
Et	V_2_O_5_	Thin films	573–773	1.27–1.80	500–3000	NA	[43]
Et	V_2_O_5_	Nanowiremicroyarns	600	9.09	50–1000	Higher alcohols	[44]
C_4_H_10_S Tert-butyl mercaptan	V_2_O_5_	Thick layer (0.2 mm) from nanopowder	600	Catalumine- scence	3600–62,000	Alcohols, alde-hydes, NH_3_	[45]
NO_x_ , H_2_	V_2_O_5_ + VO_x_	Thin film composed from nanotubes	448 563	2.85 1.075	20–80 NO 500–2000 H_2_	CO	[46,47]
CH_4_	Au–VO_x_	Porous thin film	RT	NA	1500	NA	[48]
CH_4_	C/VO_x_	C nanotubes filled with VO_x_	RT	1.015	NA	NA	[49]

Symbols: Et^1^—ethanol (C_2_H_5_OH); NA^2^—not reported; RT^3^—room temperature (298 K); BTEX^4^—benzene, toluene, ethylbenzene and xylene.

**Table 2:** Vanadium oxide-based semiconducting NO_2_ sensors.

Materials	Operation Temperature [K]	Concentration	Response	Reference
VO_2_ thin film nanocolumnar	423	>100 ppm	5	[24]
V_2_O_5_ thin film nanotubes	563	20–80 ppm	6	[47]
V_2_O_5_ thin film nanotubes	448	20–80 ppm	2.9	[47]
Composite porous Si/ nanorods V_2_O_5_	298–523	0.25–3	5–10	[51]
V_2_O_5_ thin film	553–573	100 ppm	1.6	[62]
V_2_O_5_ thin films composed from-nanorods	473	100 ppm	1.24	[63]
V_2_O_5_ thin films 450 nm	323	2–20 ppm	1.8	[64]
V_2_O_5_ nanorods	473	100 ppm	1.75 1.24	[65] [66]
V_2_O_5_ thin films	410–545	4 ppm 20 ppm	1.16 3.35	This work
V_2_O_5_ thin films	546–617	4 ppm 20 ppm	14.4 23.0	This work
